# Three-dimensional chromatin architecture datasets for aging and Alzheimer’s disease

**DOI:** 10.1038/s41597-023-01948-z

**Published:** 2023-01-24

**Authors:** Guofeng Meng, Hong Xu, Dong Lu, Shensuo Li, Zhenzhen Zhao, Haohao Li, Weidong Zhang

**Affiliations:** 1 Institute of interdisciplinary integrative Medicine Research, Shanghai University of Traditional Chinese Medicine, Shanghai, 201203 China; 2 Faculty of Business and Economics, The University of Hong Kong, Pokfulam Road, Hong Kong, China

**Keywords:** Data processing, Data acquisition

## Abstract

Recently, increasing studies are indicating a close association between dysregulated enhancers and neurodegenerative diseases, such as Alzheimer’s disease (AD). However, their contributions were poorly defined for lacking direct links to disease genes. To bridge this gap, we presented the Hi-C datasets of 4 AD patients, 4 dementia-free aged and 3 young subjects, including 30 billion reads. As applications, we utilized them to link the AD risk SNPs and dysregulated epigenetic marks to the target genes. Combining with epigenetic data, we observed more detailed interactions among regulatory regions and found that many known AD risk genes were under long-distance promoter-enhancer interactions. For future AD and aging studies, our datasets provide a reference landscape to better interpret findings of association and epigenetic studies for AD and aging process.

## Background & Summary

Alzheimer’s disease (AD) is a prevalent neurodegenerative disorder among the aged population. The main clinical features include memory and learning deficits, disorientation, mood swings, and behavioral issues^1^. Studies of patients with familial (early-onset) AD identified autosomal dominant mutation of the amyloid precursor protein (*APP*), presenilin 1, and presenilin 2^2^. However, these mutations account for only 1%–5% of the total disease burden^3^. Most cases of AD are late-onset (>65 years), which are caused by complex crosstalk of genetic and environmental factors^4,5^. Genome-wide association studies have identified many risk genes^6–9^. These genes function in diverse biological processes, such as immune system process (*TNF*, *IL8*, *CR1*, *CLU*, *CCR2*, *PICALM*, and *CHRNB2*), cellular membrane organization (*SORL1*, *APOE*, *PICALM*, *BIN1*, and *LDLR*), and endocytosis (*PICALM*, *BIN1*, and *CD2AP*)^10^. However, the identified AD risk-associated genes only contribute to a small portion of AD pathogenesis^11^, thus limiting their application in causal mechanism studies and new drug discovery^12^. For sporadic AD, age is the biggest risk factor for AD genesis^13^. Studies suggest that AD and aging are intrinsically interwoven with each other^14,15^. For example, brains of elder individuals contain abnormal deposits of aggregated proteins such as hyperphosphorylated tau (p-tau), amyloid-*β* (A *β*), and *α*-synuclein^16^; however, it remains unclear whether they are linked to AD genesis. For AD studies, an open question is if there is any molecular mechanism, especially aging-related mechanism, mediating these diverse biological processes.

Recently, an increasing number of studies have revealed the importance of the dysregulation occurring in cis- or trans-regulatory regions. Expression quantitative trait loci (eQTL) analysis supports the proposition that AD risk-associated single nucleotide polymorphisms (SNPs) take regulatory roles by affecting the expression of nearby AD genes in the form of looped interactions^6,17–19^. Large-scale DNA methylation studies have identified hypo- and hyper-methylated enhancers in postmortem AD brain samples, which alter the regulation of AD risk genes^20–22^. H3K27ac, a marker for active enhancers and promoters, is differentially distributed at the regulatory regions involved in the progression of amyloid-*β* and tau pathology^23^. Epigenetic studies on other histone marks, for example, H3K9me3^24^, H3K9ac^25^, and H4K16ac^26^, have also revealed critical links from epigenomic dysregulation to AD genesis. Meanwhile, studies of epigenetic drugs have suggested the benefits of epigenetic modification. For example, inhibition of the HDAC3 protein by RGFP966 can reverse AD-related pathologies *in vitro* and *in vivo* mouse models^27^. In our previous study using large-scale AD patients, we predicted that AD patients suffer from transcription regulation degeneration, which disrupts many AD-related pathways^28^. However, it is still not clear how the changes in the non-coding regulatory regions, especially epigenetic changes, contribute to AD genesis. It requires a detailed map of long-distance interaction to link regulatory regions to disease genes^29^.

**Fig. 1:**
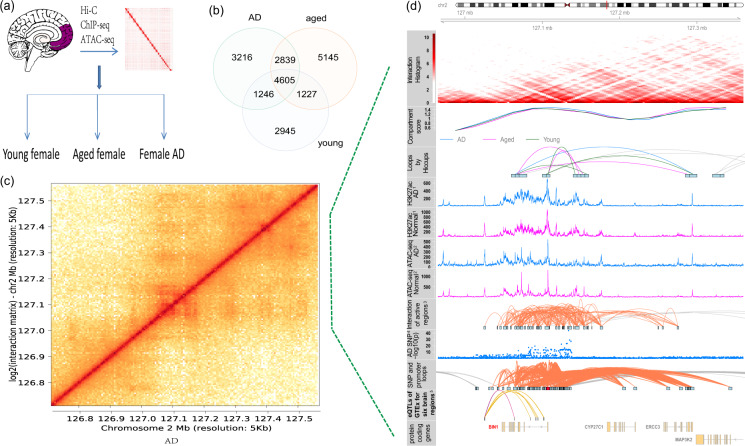
Three-dimensional chromatin architecture datasets for aging and Alzheimer’s disease. (**a**) Post-mortem brain tissue from female AD patients and cognitively normal elderly and females were studied using Hi-C; other data were also generated or collected to facilitate the understanding of Hi-C results, e.g., ATAC-seq, H3K27ac ChIP-seq, and GWAS SNPs. (**b**) The loops identified by HiCCUPS for three groups of samples. (**c**) Chromosome interactions heatmap for an exemplary region around *BIN1* at chr2 of AD sample. (**d**) Integrative analysis results, including an interaction histogram, compartment score, loops predicted by HiCCUPS, H3K27ac peaks, open chromatin regions by ATAC-seq, contacts of active regions, AD risk SNPs, SNP-promoter interactions, eQTLs, and protein-coding genes. The height of loops indicated the log2 transformed contact frequency. Note that eQTLs of non-coding genes were also displayed in the eQTL track and no eQTL was identified for the BIN1 gene at *p* < 1*e*-5. Note that the used data are from different sources. Track 1 from entorhinal cortex samples of AD cases and matched controls [23]; Track 2 from prefrontal cortex region of Chinese AD samples; Track 3 is inferred regions by integrative analysis of Track 1 and Track 2; Track 4 is collected from a published meta-analysis on PGC-ALZ, IGAP, ADSP, and UKB^8,9^; Track 5 are downloaded from the GTEx database (https://gtexportal.org/).

In this dataset, we generated high-resolution maps of three-dimensional (3D) chromatin architecture of aging and AD using Hi-C technology. The prefrontal cortex region of post-mortem brain tissue from dementia-free elderly females (hereafter called “aged”, n = 4, mean age = 90, Chinese), female patients with AD (n = 4, mean age = 91.5, Chinese), and cognitively normal young females (hereafter called “young”,n = 3, mean age = 29, Chinese) were used for Hi-C sequencing (see Fig. 1a and Table 1). All the selected AD samples were carefully evaluated so that AD patients all had similar and severe disease conditions; aged and young samples were free of dementia. During the sequencing step, two samples were randomly selected from the aged and AD groups to generate 800 million paired-end reads with an estimated resolution of 9000 bp. The other samples were sequenced for 3 billion reads with an estimated resolution of 3000 bp. After merging Hi-C data from the same group, the HiCCUPS tool identified 11906, 13816, and 10023 loops at a cutoff of FDR <0.1 for AD, aged, and young groups, respectively (see Fig. 1b). To perform integrative analysis, other data were also generated or collected to facilitate the understanding of Hi-C results, e.g., ATAC-seq, H3K27ac ChIP-seq, and GWAS SNPs. As an example, Fig. 1c,d shows the integrated results for *BIN1* gene and the surrounding regions. Compared with nearby genes, more Hi-C loops, H3K27ac marks, ATAC-seq signals, and AD GWAS SNPs are observed from upstream to gene body of *BIN1*, indicating that *BIN1* is under intensive regulation. The Hi-C analysis results are also presented, including interactions of active regulatory regions and SNP-promoter interactions. This result indicates that long-distance interactions are closely related to *BIN1* activities. The analysis results for more genes and genomic regions are available in http://menglab.pub/hic.

**Table 1:** Sample information for 11 samples.

No.	ID	Random Label	Status	Est. Resolution	gender	age	Braak NFT stage	PMI(Min)	CSF PH	No.reads/billion	No.nodup.Validated Pairs/billion
1	sample1	smp1	aged	8700	female	90	0	300	7.0	0.71	0.26
2	sample2	smp2	AD	8250	female	86	6	120	7.0	0.72	0.25
3	sample3	smp3	young	3200	female	28	0	300	7.2	3.15	1.07
4	sample4	smp4	AD	3200	female	91	6	210	7.0	2.98	1.07
5	sample5	smp5	young	3100	female	30	0	260	7.3	2.80	1.07
6	sample6	smp6	young	2650	female	29	0	310	7.0	3.03	1.31
7	sample7	smp7	AD	3750	female	94	5	240	7.0	2.94	0.99
8	sample8	smp8	aged	3800	female	91	1	241	-	3.30	0.98
9	sample9	smp9	aged	3200	female	94	0	240	8.3	3.00	1.16
10	sample10	smp10	AD	3200	female	95	5	180	7.0	2.59	1.05
11	sample11	smp11	aged	3200	female	85	0	200	7.0	2.10	1.04

## Methods

### Sample collection for Hi-C study

Postmortem brain samples (prefrontal cortex regions) of 11 female individuals, including 4 subjects diagnosed with AD (Braak NFT stage >4, mean age = 91.5), 4 age-matched normal subjects (mean age = 90), and 3 young subjects (mean age = 29), were collected from the Chinese Brain Bank Center in Wuhan (CBBC, http://cbbc.scuec.edu.cn) and China Brain Bank, Zhejiang University (http://www.neuroscience.zju.edu.cn). Informed consent for autopsy had been provided by all subjects during life. This study was reviewed and approved by the Ethics Committee of both brain banks and Shanghai University of Chinese Medicine. The clinical information of each subject was reviewed by independent neurologists with expertise in dementia, and the neuropathological diagnosis was given regarding the most likely clinical diagnosis at the time of death. AD samples were carefully evaluated so that all included AD subjects with homogeneous disease status. The following criteria are more considered: (1) Braak NFT stage of ≥5 and severe disease stages; (2) within an age range of 85 to 95; (3) no or weak neuronal loss; and (4) not affected by other neurological diseases.

#### Hi-C

The AD, aged, and young samples were randomly labeled, and the sample information was blind to experimental staff. The Hi-C experiment was performed following the protocol introduced in^30^. Brain cells were suspended in lysis buffer (10 mMTris, pH 8.0, 10 mM NaCl, 0.2% Igepal CA-630 and 1 × cOmplete^*TM*^ protease inhibitors (Sigma-Aldrich, 11697498001) and incubated on ice for 10 mins. Centrifugation at 2500 g for 5 mins at 4 °C, followed by removal of supernatant. Resuspended in 342 *μ*L 1 × NEBuffer 3.1, and incubated with 38 *μ*L 10% SDS at 65 °C for 10 mins. Added 43 *μ*L of 10% Triton X-100 to the Hi-C-tube to quench the SDS at 37 pellet for 15 min. Added 12 *μ*L 10 × NEBuffer 3.1 and 400U DpnII (NEB, R0543), and mixed to digest the chromatin overnight at 37pellet on a rocking platform. Inactivated DpnII restriction enzyme at 65 °C for 25 mins. Then, biotin-14-dATP (Life Technologies, 19524-016), dCTP, dGTP, dTTP and DNA polymerase I Kenow were added (NEB, M0210), and incubated at 23 °C for 4 h. The digested chromatin was diluted and re-ligated by T4 DNA ligase (NEB, M0202), incubated at 16 °C for 4 h, and shaken for three times. De-cross-linked by adding 30 *μ*L proteinase K, and incubated at 65 °C overnight. The DNAs were extracted and dissolved in 50 *μ*l 10 mMTris, pH 8.0. Then T4 DNA polymerase (NEB, M0203) was added and removed biotin for 4 hr at 20 °C, and the enzymes were inactivated for 20 mins at 75 °C. The DNAs were sheared to a size of 300 bp using Covaris M220. Pulldown with Streptavidin T1 beads (Life Technologies, 65602). Then, performed end repair, A adding, adaptor adding reaction, PCR amplification and DNA products size selection. The libraries were sequenced by the Illumina NovaSeq. 6000 sequencing platform.

### Hi-C data analysis

The raw sequencing data were cleaned with the trimmomatic tool under the default setting^31^. The cleaned fastq data were input to HiC-Pro pipeline^32^ to generate non-duplicated valid pairs, and we recorded the genomic interactions reported by ligated reads. The UCSC hg38 genome was used for alignments. The quality of analysis results in each step was evaluated following the protocol introduced in https://www.encodeproject.org/pipelines/, including inter-/intra-chromosomal pairs, chimeric pairs, duplicates, intra-fragment, intra-long distance ranges, and ligations. The sparse interaction matrices were generated at different bin sizes, ranging from 2000 to 200,000 bp. The compartment discovery and differential compartment activity analysis were performed using HOMER^33^ with a bin size of 25,000 bp under the default parameter setting. The first principal component (PC1) of principal component analysis (PCA) was used to indicate compartment A/B along the genome. During this step, the samples from paired groups were input for differential compartment activity analysis. To avoid the flipped signs of PC1 values, we applied two steps: (1) we compared the signs of each bin across the samples of the same groups; (2) we used H3K27ac signals to decide PC1 signs of bins. The PC1 value along the hg38 genome was recorded in bedGraph format for visualization.

### Loops discovery analysis

The resolution of Hi-C data of each sample was estimated by applying juicer tool. We found that the resolution of 9 samples with higher sequencing depth was about 3200 bp and two samples had a resolution of 8500 bp. The loops were predicted using HiCCUPS^34^ at a cutoff of FDR <0.1 at two bin sizes of 5000 and 10000 bp, respectively. To further improve the resolution, the valid pairs generated by HiC-Pro tool from the same sample group were merged together and then transformed into *.hic files. The loops were predicted using HiCCUPS under default parameter settings. By analyzing the HiCCUPS output, many loops were reported in only one or two groups. We checked the contacting frequency for these group-specific loops in other groups and did not find any loop with completely loop loss or gain in all three groups. Therefore, the loops identified in AD, aged, and young samples were merged into non-overlapped ones. In this process, bedtools^35^ was used by setting the minimum overlap as 5000 bp or the max length of loop anchors, and ensuring that there was zero gap. The self-contacted loops were filtered so that the anchoring regions of the same loops were not overlapped^36^.

### TADs discovery analysis

Topologically associating domains (TAD) were discovered with HOMER using the script of findTADsAndLoops.pl^33^. This tool works by generating relative contact matrices for each chromosome and scanning them for locally dense regions of triangle domains that have a high degree of inter-domain interactions relative to their surrounding region. In this step, we set the resolution to 3000 bp and an overlapping window size of 15000 to find the TADs.

### Normalized contact matrices

The raw contact matrices were generated by Hic-Pro at an arbitrary bin size of 5000 bp or 10000 bp. To generate comparable contact matrices, the raw interaction matrices of 11 samples were normalized using the R tool multiHiCcompare^37^. We firstly filtered the interactions with a total frequency of less than 20. In this step, only the intra-chromosomal interaction was considered. Under the default parameter setting, a normalized contact matrix for each chromosome was generated under default parameter setting. To evaluate the quality of Hi-C data, we performed clustering analysis, including principal component analysis and hierarchical clustering, using all whole interaction profiles. Our analysis found that sequencing depth or ratio of uniquely mapped reads had a significant impact on the output matrices. Similar results were also observed with the reproducibility analysis using the raw matrix data^38^. Therefore, the normalized interaction matrices were adjusted to remove their effects using the ComBat tool in R package sva^39^. In this process, 11 samples were classified and labelled as high-resolution and medium-resolution samples; the ratios of uniquely mapped reads were treated as a continuous covariate.

### Mapping regions to the gene body

The anchor regions of loops or differential interactions were mapped to the gene bodies by R package ChIPseeker^40^. The gene body annotation was based on known genes from UCSC build hg38, including promoter, 5′UTR, 3′UTR, exon, intron, downstream, and intergenic regions. The promoter regions were defined as regions from −2000 bp to 2000 bp around TSS. Most human genes have multiple promoters, and these promoters were all considered.

### Assay for Transposase-Accessible Chromatin using sequencing (ATAC-seq)

ATAC-seq was performed in GENEWIZ company following the protocol introduced in^41,42^. Postmortem brain samples in the prefrontal cortex regions of 26 individuals, including 13 diagnosed with AD and 13 normal subjects were collected from the Chinese Brain Bank Center in Wuhan (CBBC, http://cbbc.scuec.edu.cn) and China Brain Bank, Zhejiang University (http://www.neuroscience.zju.edu.cn). Then, place frozen tissue into a pre-chilled 2 ml Dounce with 2 ml cold nuclei lysis buffer. Allow frozen tissue to thaw for 5 minutes. Dounce with A pestle until resistance goes away (10 strokes). Dounce with B pestle for 20 strokes. Pre-clear larger chunks by pelleting at 100 RCF for 1 min in a pre-chilled centrifuge.Count nuclei using Trypan blue staining and aliquot nuclei for ATAC reaction. Harvest and count cells. Cells should be intact and in a homogenous, single-cell suspension; Centrifuge 50,000 cells 5 min at 500 × g, 4 °C. The number of cells at this step is crucial, as the transposase-to-cell ratio determines the distribution of DNA fragments generated. Remove and discard supernatant. Wash cells once with 50 *μ*l of cold PBS buffer. Centrifuge 5 min at 500x g, 4 °C. Remove and discard supernatant. Gently pipet up and down to resuspend the cell pellet in 50 *μ*l of cold lysis buffer. Centrifuge immediately for 10 min at 500 × g, 44 °C. Discard the supernatant, and immediately continue to transposition reaction. Make sure the cell pellet is set on ice. To make the transposition reaction mix, combine the following: TD (2x reaction buffer from Nextera kit) 25 *μ*l; TDE1 (Nextera Tn5 Transposase from Nextera kit) 2.5 *μ*l; Nuclease-free H2O 22.5 *μ*l. Resuspend nuclei pellet in the transposition reaction mix. Incubate the transposition reaction at 37 °C for 30 min. Gentle mixing may increase fragment yield. Immediately following transposition, purify using a Qiagen MinElute PCR Purification Kit. Elute transposed DNA in 10 *μ*l Elution Buffer (Buffer EB from the MinElute kit consisting of 10 mM Tris·Cl, pH 8). To amplify transposed DNA fragments, combine the transposed DNA (10 *μ*l), nuclease-free H2O (10 *μ*l),25 *μ*M PCR Primer 1 (2.5 *μ*l), 25 *μ*M Barcoded PCR Primer 2 (2.5 *μ*l), NEBNext High-Fidelity 2x PCR Master Mix (2 5 *μ*l). Thermal cycle as follows 72 °C,5 min, 1 cycle; 98 °C,30 sec; 98 °C, 10 sec 5 cycles; 63 °C, 30 sec; 2 °C, 2 min; 4 °C.

### Cis-regulatory regions

In this work, we used H3K27ac and ATAC-seq signals to define cis-regulatory regions (CREs). Raw data of H3K27ac ChIP-seq were collected from the GEO database with ID of GSE102538^23^, where 47 post-mortem entorhinal cortex tissue samples were used to identify widespread AD-associated acetylomic variations. Cleaned fastq files were aligned to the human genome hg38 following the instructions of the original paper. In this step, duplicated reads were removed. The sorted and indexed bam files were merged together by samtools^43^ into a single bam file, and then we performed peak calling using macs2 under a parameter setting of “–keep-dup all–broad–broad-cutoff 0.1”. ATAC-seq data were generated and analyzed as described in our previous work^28^, including the prefrontal cortex regions of 13 Chinese people with diagnosed with AD and 13 Chinese normal subjects. Like ChIP-seq data, AD and normal samples were merged for peak calling with a parameter setting of “–keep-dup all–nomodel–shift −100–extsize 200”. We applied bedtools to check the peak overlaps of H3K27ac ChIP-seq and ATAC-seq. The peaks for H3K27ac marks and ATAC-seq were merged into non-overlapped regions, which were treated as active regions of the brain. We mapped these regions to gene bodies and classified them as promoters, enhancers, and other regions. The active regions that locate in the regulatory region, are treated as CREs.

### AD risk SNPs

The AD GWAS analysis results were collected from a recently published meta-analysis on PGC-ALZ, IGAP, ADSP, and UKB^6–9^. The SNPs and their significance were downloaded from https://ctg.cncr.nl/software/summary_statistics. The AD risk SNPs were selected with a cutoff of *p* < 1*e*-5, and their genomic locations were transformed into the corresponding locations on hg38 genome. In total, 6468 SNPs were selected to check their overlap with Hi-C interactions and active regions or enhancers.

### Hi-C loops of SNP-promoter interactions

The genomic locations of AD risk SNPs were mapped to the human genome based on the annotation of dbSNP database. We filtered the SNPs located in the promoter regions (±2000 bp around TSS). The TSS information was collected from the ENSEMBL database and the promoter regions were defined as from −2000bp to 2000 bp around TSS. Most human genes have multiple promoters, and these promoters were all considered. Next, we identified the loops linking SNPs to promoters. It is known that the functional SNPs in the noncoding regions usually take roles by affecting the transcription factor binding. However, there is no golden standard to define the ranges of cis-regulatory regions. Here, we arbitrarily set a region of 1000 bp around SNPs as the SNP-affected cis-regulatory regions. The contacting frequency between promoter and SNP regions was calculated by *bedtools findoverlaps*, which counted the number of reads that anchored at both SNP and promoter regions. To find a proper cutoff of contacting frequency, we calculated the frequencies of all possible SNP-promoter pairs on the same chromosome and we found that most of the pairs have a contact frequency of 0. Among the pairs linked by at least one Hi-C read, there are less than 1% of SNP-promoter pairs with frequency >20 (*p* < 0.01). If the contacting frequency was great than 20, the corresponding SNP-promoter regions were supposed to be linked by loops. Additionally, we also performed loop discovery using bin-free tool Binless^44^ under the suggested parameter setting.

For interaction profiles of active regions, we applied R package InteractionSet to calculate the interaction frequency between active regulatory regions. The pairs with less than 20 interactions or spanning different chromosomes were filtered.

### eQTL for human brain regions

The significant eQTLs were downloaded from the GTEx database (https://gtexportal.org/), where eQTLs had been filtered at a cutoff of FDR <0.05. Based on text mining, we selected eQTLs of six brain regions: the amygdala, anterior cingulate cortex, cortex, frontal cortex, hippocampus, and hypothalamus. As an independent validation, we also included the eQTLs stored in BRAINEAC database (http://www.braineac.org/)^45^ to evaluate the interactions reported by GTEx or Hi-C datasets.

### MSBB data analysis

The RNA-seq and clinical data of AD patients were downloaded from the AMP-AD project https://www.synapse.org/#!Synapse:syn2580853. To simplify the analysis, only MSBB data were selected. After filtering the sample with incomplete information, MSBB data included 223 AD patients and four brain regions: the frontal pole (BA10), superior temporal gyrus (BA22), parahippocampal gyrus (BA36), and frontal cortex (BA44). These subjects had diverse clinical manifestations, for example, cognitive score and Braak stages. Approximately 61% were diagnosed as having pathological AD or probable AD. The clinical dementia rating scale (CDR) and mini–mental state examination (MMSE) severity tests were used to assess cognitive status. Based on CDR classification, subjects were grouped as no cognitive deficits (CDR = 0), questionable dementia (CDR = 0.5), mild dementia (CDR = 1.0), moderate dementia (CDR = 2.0), and severe to terminal dementia (CDR = 3.0–5.0). The differential expressed genes were identified by the R limma package, where two conditions were compared for all the expressed genes.

### Visualization of long-distance interaction

The interaction matrices for AD, aged, and young groups were generated by merging the matrices from different samples. Then the interaction matrices were normalized by the Knight-Ruiz (KR) method^37^. An interaction heatmap was generated by HiCPlotter^46^. R package Gviz was used to visualize the binned triangle interaction heatmap, compartment A/B, H3K27ac ChIP-seq, ATAC-seq, GWAS SNP significance signal, and mapped genes along the human genome. The contacting loops were mapped by R package GenomicInteractions^47^.

## Data Records

The Hi-C raw fastq files were deposited at NCBI under accession number of SRP280183^48^. The raw ATAC-seq data were publicly available in the Gene Expression Omnibus (GEO) database with the ID of GSE129041^49^. To facilitate the usage of Hi-C datasets, we build a shiny-based tool at http://menglab.pub/hic/. It can perform integrative analysis for genes or user-defined genomic regions, including an interaction histogram, compartment score, loops predicted by HiCCUPS, H3K27ac peaks, open chromatin regions by ATAC-seq, contacts of active regions, AD risk SNPs, SNP-promoter interactions, eQTLs, and protein-coding genes. The processed Hi-C data are also available for public download in the same web tool.

## Technical Validation

### Quality assessment of Hi-C data

**Fig. 2:**
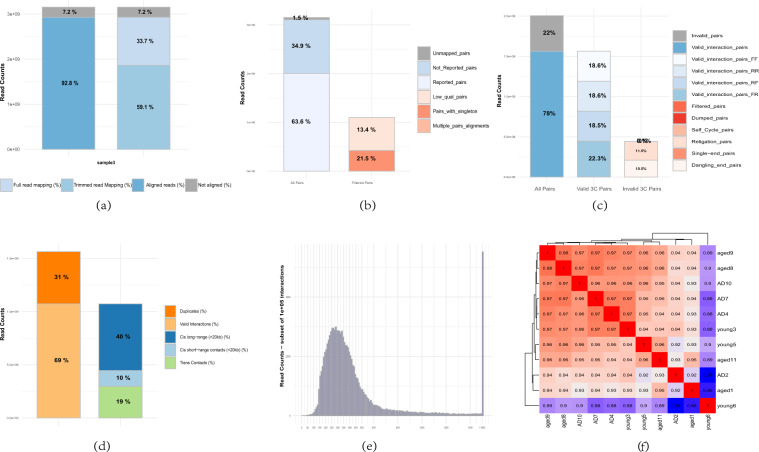
Quality assessment of Hi-C datasets. (**a**) Statistics of reads alignment. (**b**) Statistics of reads pairing. (**c**) Statistics of read pairs alignment on restriction fragments. (**d**) Statistics of validated pairs and interaction types. (**e**) Fragment size distribution of valid pairs. (**f**) Reproducibility analysis using HicRep for 11 samples.

Quality assessment of each Hi-C dataset was performed following the protocol introduced in https://www.encodeproject.org/pipelines/, including inter-/intra-chromosomal pairs, chimeric pairs, duplicates, intra-fragment, intra long distance ranges, and ligations (see here^50,51^). Figure 2a shows the results of reads mapping step using one example of AD samples. >90% of reads were aligned on the genome of hg38 by bowtie2^52^. Figure 2b shows the pairing statistics by HiC-Pro, in which more than 60% reads were paired. Among the pairs, more than 70% are validated pairs (see Fig. 2c). Among the validated pairs, there are about 30% of duplicates, which is mainly due to the high sequencing depth (see Fig. 2d). Fragment size distribution was extracted from the valid interaction and we observed a distribution centered around 300 bp, which corresponds to the paired-end insert size commonly used (see Fig. 2e). For the other samples, we also perform the same quality assessment and observed similar results^50^. We also performed reproducibility analysis by HICRep tool^38^ among samples. We found that the samples had overall good similarity (>0.85) in interaction profiles (see Fig. 2f). Overall, all the evaluation results suggested a good quality of the Hi-C data.

### Hi-C interaction sites overlap with promoters and enhancers

**Fig. 3:**
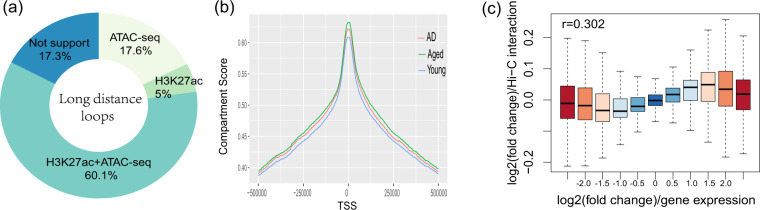
Hi-C interaction sites overlap with promoters and enhancers. (**a**) Graph illustrating that 82.7% of loops were overlapped with active chromatin regions, which were defined by H3K27ac marks and ATAC-seq peaks.(**b**) Compartment analysis indicated that the transcription start sites were more associated with active looped interactions. (**c**) Chromatin-looped interactions were correlated with the gene expression changes. Boxplot analysis showed the fold changes of long-distance interactions and gene expression, indicating that the dynamic of 3D-chromatin architecture contributed to gene expression changes.

For the Hi-C loops, we found that 60% of them were overlapped by both H3k27ac and ATAC-seq peaks (see Fig. 3a). We also checked the genomic distribution of anchor sites of Hi-C loops and found that more than 40% of the loops overlapped with gene promoter regions within 1000 bp around transcription start sites (TSS). Moreover, 18% of the loops were mapped to the distal intergenic region^50^. Compartment A/B analysis indicated that the TSS regions were more active in compartment activity (see Fig. 3b). All of these results suggested that Hi-C loops were more related to active regulatory regions on the chromatins.

We investigated the relationship among long-distance interaction, chromatin status, and gene expression. Hence, we checked whether long-distance interactions contributed to gene expression regulation using a method introduced in^53^. We identified 2704 differentially expressed genes (DEGs) in AD patients using the MSBB dataset from AMP-AD projects. We found that fold changes of DEGs had a good correlation with the changes in long-distance interactions, and the Spearman’s correlation was 0.3 (*p* = 2.36*e*-57, see Fig. 3c), which is comparable to findings in neuron cells^53,54^.

### Link AD risk SNPs to risk genes

**Fig. 4:**
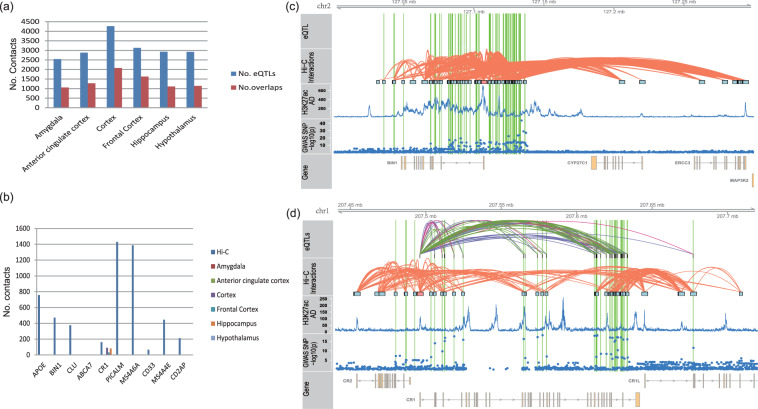
Links of AD risk SNPs to target genes. (**a**) The significant eQTLs collected from the GTEx database for six brain regions and their overlaps with Hi-C loops. Here, eQTLs were selected at a cutoff of FDR <0.05. (**b**) More SNP-promoter links were identified by Hi-C loops than by eQTL. Using the top 10 AD risk genes as an example, SNP-promoter links were identified for nine out of ten AD risk genes by Hi-C loops, whereas only one gene was identified by eQTLs. (**c**,**d**) SNP-promoter links for *BIN1* and *CR1* genes. Here, both genes were heavily linked by AD-associated SNPs but no eQTL link was identified for the *BIN1* gene. The green line marks the AD risk SNPs with links to the target genes.

To build the links from AD risk SNPs to genes, 6468 AD risk SNPs were collected from published GWAS data^6^ at a cutoff of *p* < 1.0*e*-5. After filtering, 3498 SNPs located within promoter regions were selected for SNP-promoter loop discovery. We identified 75,953 SNP-promoter links with a contacting frequency of >20. They included 2771 AD risk SNPs and 355 genes^55^. We attempted to evaluate if existing brain eQTL can help to link AD-related enhancers to their target genes. Therefore, we collected 7561 significant eQTLs from the GTEx database for six brain regions (the amygdala, anterior cingulate cortex, cortex, frontal cortex, hippocampus, and hypothalamus). Among these eQTLs, 3417 were overlapped with Hi-C loops. Figure 4a shows the number of eQTLs and their overlaps with Hi-C loops in the six brain regions. Overall, a modest overlap was observed between Hi-C loops and eQTL. SNP-promoter links were then evaluated using the top 10 AD risk genes in the AlzGen database (see Fig. 4b). The promoters of nine genes were heavily contacted with the AD-associated SNPs. However, eQTLs only reported the links for one gene, *CR1*; that is, eQTL almost failed to identify the target genes of AD-associated SNPs. Taking *BIN1* as an example, there were 126 AD-associated SNPs along the gene body and upstream regions. Hi-C data supported that 63 of them were linked to the promoter of the *BIN1* gene(see Fig. 4c), which validated the roles of AD-associated SNPs in the activity of *BIN1*. Among AD-associated SNPs within or surrounding the *BIN1* gene, rs4663105 was the most associated SNP, with a significance of *p* = 1.45*e*-44. However, it had no link with the *BIN1* promoter. Similar results were observed for other AD risk SNPs, including the six most associated SNPs. Among the SNPs with Hi-C links to *BIN1*, rs35103166, an SNP located in the upstream region of *BIN1*, was associated with AD at *p* = 4.79*e*-23. The contacts between rs35103166 and *BIN1* promoters were supported by 99 reads, supporting a high-confidence interaction. Similar results were observed with other AD risk SNPs. Unlike with Hi-C loops, we did not find any eQTL link between AD-associated SNPs and *BIN1* expression. We also checked the eQTLs reported by BRAINEAC database^45^ and did not find eQTL links to *BIN1* under a cutoff of *p* < 0.01. Another example can be seen with *CR1* (see Fig. 4d), which was linked by eQTLs of multiple AD-associated SNPs. There were 48 AD-associated SNPs near or within the *CR1* gene, among which 37 SNPs were linked to *CR1* by 67 eQTLs in the six brain regions. Most of these SNP-gene links were supported by Hi-C SNP-promoter loops. Hi-C loops reported more SNP-promoter links for 43 AD risk SNPs.

### Integrating hyper-acetylated peaks to identify the dysregulated enhancer-promoter interactions

**Fig. 5:**
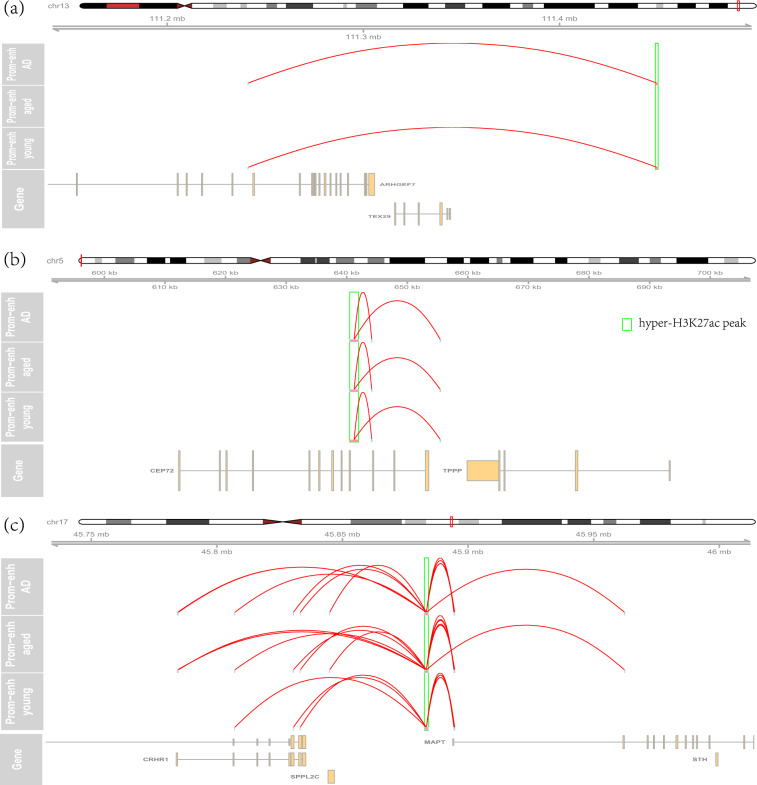
Link hyper-acetylated peaks to the target genes. (**a**) The long-distance loops link the most hyper-acetylated peak to its target gene, *ARHGEF7* gene. (**b**) The long-distance loops link the top-ranked hyper-acetylated peak on chr5:640598–642071 to CEP72 gene. (**c**) The long-distance loops link the hyper-acetylated peak to the promoter of *MAPT* and *CRHR1* gene.

The expensive cost of Hi-C experiments limits its application to too many samples. To identify the changed enhancer-promoter regulation, a feasible solution is to integrate Hi-C annotation with other dysregulated signals. As a demonstration, we collected 1475 hyper-acetylated H3K27ac peaks from the published study^23^ and identify the target genes of 806 peaks^56^. Figure 5a shows the results of a peak on chr13:112101248–112102698, which was reported as the most hyper-acetylated peak. This peak located at the downstream of *ARHGEF7* and *TEX29* genes. It interacted with the promoter of ENST00000483189, one transcript of *ARHGEF7* gene. This result suggested that *ARHGEF7* might take a more important role in AD. It should be noticed that *ARHGEF7* was not the proximal gene according to their genomic location, which suggested a necessity to use Hi-C results to annotate dysregulated regulatory elements. Figure 5b shows another example of the most hyper-acetylated peak on chr5:640598–642071, located in the ninth intron of *CEP72*. It interacted with the promoter of ENST00000512038, a transcript of *CEP72*. Under our parameter setting, we failed to identify links from some top-ranked hyper-acetylated peaks and it is not clear if they are involved in transcription regulation.

One of the hyper-acetylated peaks of chr17:43959954–43961546 is proximal to the *MAPT* gene. Figure 5c shows its chromatin interaction with *MAPT* transcript promoters. Multiple *MAPT* transcripts were linked to this peak, e.g. ENST00000571311, ENST00000420682, and ENST00000262410. Additionally, the same peak is also linked to the promoters of multiple transcripts of *CRHR1* gene. *CRHR1* has been reported for association with synaptic loss and memory in other neurological diseases^57^. This result might indicate its potential involvement in AD.

## Data Availability

The Hi-C data analyses were performed using public tools. The following softwares were used to perform Hi-C data analysis: 1. FastQC v0.11.9 https://www.bioinformatics.babraham.ac.uk/projects/fastqc/ 2. HiC-Pro v2.11.3 https://github.com/nservant/HiC-Pro 3. Juicer tools v1.14.08 https://github.com/aidenlab/Juicebox 4. HOMER V2.0 http://homer.ucsd.edu/homer/interactions2/index.html 5. bedtools v2.29.2 https://bedtools.readthedocs.io/en/latest/ 6. Gviz V1.40.0 https://bioconductor.org/packages/release/bioc/html/Gviz.html 7. ChIPseeker v1.32.0 http://bioconductor.org/packages/release/bioc/vignettes/ChIPseeker 8. HiCPlotter v0.6.6 https://github.com/akdemirlab/HiCPlotter 9. multiHiCcompare v1.14.0 https://dozmorovlab.github.io/multiHiCcompare/ 10. MACS v2.2.6 https://github.com/macs3-project/MACS 11. R v3.6.2 https://cran.r-project.org/ 12. limma v3.1.2 https://bioconductor.org/packages/release/bioc/html/limma.html
